# Ultra-hypofractionated radiotherapy with focal boost for high-risk localized prostate cancer (HYPO-RT-PC-boost): *in silico* evaluation with histological reference

**DOI:** 10.2340/1651-226X.2025.44211

**Published:** 2025-10-27

**Authors:** Erik Nilsson, Anneli Nilsson, Joakim Jonsson, Kristina Sandgren, Josefine Grefve, Jan Axelsson, Angsana K. Lindberg, Karin Söderkvist, Camilla T. Karlsson, Björn Zackrisson, Sara Strandberg, Katrine Riklund, Anders Bergh, Mathieu Moreau, Adalsteinn Gunnlaugsson, Lars E. Olsson, Tufve Nyholm

**Affiliations:** a Department of Diagnostics and Intervention, Radiation Physics, Umeå University, Umeå, Sweden; b Department of Diagnostics and Intervention, Oncology, Umeå University, Umeå, Sweden; c Department of Diagnostics and Intervention, Diagnostic Radiology, Umeå University, Umeå, Sweden; d Department of Medical Biosciences, Pathology, Umeå University, Umeå, Sweden; e Department of Hematology, Oncology and Radiation Physics, Skane University Hospital, Lund University, Lund, Sweden; f Department of Translational Medicine, Medical Radiation Physics, Lund University, Malmö, Sweden

**Keywords:** In silico, radiotherapy, hypofractionation, focal boost, prostate cancer

## Abstract

**Background and purpose:**

The study aims to evaluate dosimetric properties of hypofractionated treatment plans integrating focal boost, using registered whole-mount histopathology (WMHP) as reference standard.

**Methods:**

Fifteen men from the PAMP trial (EudraCT: 2015-005046-55) were included. Participants had ≥ 1 ISUP Grade group ≥ 4 lesion and underwent [^68^Ga]prostate-specific membrane antigen (PSMA) positron emission tomography/multiparametric magnetic resonance imaging (PET/mpMRI) and [^11^C]Acetate-PET/computed tomography before radical prostatectomy. Four radiation oncologists delineated gross tumor volumes (GTVs) on PSMA-PET/mpMRI. Sixty treatment plans were optimized, one per GTV and patient. Prostate planning target volumes were prescribed 42.7 Gy in seven fractions, with a simultaneous GTV boost up to 49.0 Gy, prioritizing organs at risk (OARs). Digital WMHP provided Gleason grading and was co-registered with in-vivo imaging. Target coverage for GTVs and voxels sharing Gleason patterns (GPs) was assessed via dose-volume histogram (DVH) analysis. Interobserver agreement in GTV-delineations was quantified with Fleiss’ kappa.

**Results:**

The median GTV dose per plan (D_50_) ranged from 48.3 to 49.1 Gy. For voxels with the highest GP, D_50_ was 42.9–49.2 Gy, exceeding 47.2 Gy in all except one plan. In lowest pattern voxels, D_50_ was 42.5–49.3 Gy, and below 43.4 Gy in over half the plans. Significant positive correlations between Fleiss’ kappa and DVH parameters appeared only for GP 5 regions, specifically for Fleiss’ kappa and D_50_ for two observers and the average D_50_ across observers.

**Interpretation:**

The histologically confirmed tumor was only partially boosted. Regions with more aggressive disease received better coverage. These findings provide a rational for prioritizing OARs in treatment planning.

## Introduction

Prostate cancer (PCa) tumors appear sensitive to changes in fractionation schedules, more so than many other cancers [1, 2]. These observations motivated randomized trials comparing hypofractionated treatment schedules with conventional fractionation, demonstrating similar late toxicity levels without compromising biochemical relapse-free survival [3, 4]. In addition to fractionation sensitivity, the dose–response of PCa allows for whole-gland dose escalation to improve biochemical control rates. However, this approach comes at the expense of increased toxicity levels [5–8]. Since PCa tumors often recur at the site of the primary tumor, focal dose escalation was proposed as a means to reduce biochemical recurrence without intolerable toxicity levels [9–13]. While there is limited experience in combining focal boost and hypofractionation, the results show promise [14–17].

The FLAME phase III trial showed improved biochemical control and comparable toxicity with focal boosting despite interobserver variability and institutional differences in prostate tumor contouring practices [18]. The contouring process is complicated by incomplete knowledge about the association between image- and tumor characteristics. Other challenges include lack of guidelines for boost volume delineation and dose constraints to organs at risk (OARs) in close proximity to the boost volume [12]. Previous studies have investigated the quality of gross-tumor volume (GTV) delineations on positron emission tomography (PET) and multi-parametric magnetic resonance imaging (mpMRI), by using whole-mount histopathology (WMHP) as the reference standard [19–22]. These studies show that the image modalities complement each other, but that regions with histologically confirmed PCa remain undetected. Furthermore, *in silico* planning studies with registered WMHP indicate that GTVs defined by mpMRI/PSMA-PET provide the means for focal escalation [23–25]. However, PCa is a heterogeneous disease, and it is largely unknown how the planned dose coverage varies across regions of different histological grades. This includes even the most established grading systems such as Gleason scores and ISUP grade groups (IGGs), which are relied upon due to their strong prognostic value [26, 27]. Despite these knowledge gaps, the GTV coverage in focal boosting of PCa has been associated with reduced recurrence rates [28]. Interpreting these dose–response results to accurately characterize the exposure of histologically confirmed tumor sites of varying grades remains challenging for radiotherapy (RT) patients due to the inherent lack of a gold-standard histopathological reference.

The aim of this study was to assess dose coverage across regions of varying histological grades in a hypofractionated RT regimen combined with focal boost. This was assessed by evaluating dose-volume histogram (DVH) parameters for regions defined on registered WMHP.

## Patients/material and methods

### Study population

The present study includes 15 high-risk patients (median age: 65 years; range: 54–76 years) from a larger cohort where 55 men were enrolled consecutively between December 2016 and December 2019 at the University hospital of northern Sweden (EudraCT: 2015-005046-55). Patients with elevated prostate specific antigen (PSA) (median PSA: 6.5 ng/mL; range: 3.9–13.3 ng/mL) and biopsy-proven PCa (IGG ≥ 2, at least 2 months before surgery) were planned for laparoscopic prostatectomy and underwent preoperative PSMA-PET/mpMRI and Acetate-PET/CT after providing written informed consent (Regional Ethical Board approval: Dnr 2016-220-31M). The subset of patients included in this study had at least one lesion containing an IGG ≥ 4 region, provided by WMHP. Patient characteristics are presented in Table 1.

**Table 1:** Patient characteristics.

Variable	Median (range)
Patients (*n*)	15
Age (years)	65 (54–76)
PSA (ng/mL)	6.5 (3.9–13.3)
PSA density (ng/mL²)	0.16 (0.10–0.46)
Pre-RP ISUP grade group (*n*)	
1	0
2	9
3	0
4	4
5	2
Post-RP ISUP grade group (*n*)	
1	0
2	0
3	0
4	11
5	4
pT status (*n*)	
T2a	3
T3b	12

PSA: prostate specific antigen; RP: radical prostatectomy; ISUP: international society of urological pathology; pT: pathological T-stage.. Post-RP ISUP grade group corresponds to the highest grade group recorded per patient.

### In vivo imaging

In vivo PET/mpMRI was acquired during 45 min, using an integrated 3.0 T PET/MRI system (Signa; GE Healthcare, Waukesha, WI, USA). For a detailed description of the imaging protocol, see Nilsson et al. [29] In brief, axial, coronal, and sagittal T2-weighted (T2w) imaging were obtained using fast spin-echo sequences. Apparent diffusion coefficient (ADC) images were computed using the monoexponential decay model from echo-planar diffusion-weighted imaging (DWI) with b-values of 200 s mm^-2^ and 1000 s mm^-2^. Dynamic contrast-enhanced (DCE) images were acquired by a Fast Spoiled Gradient Recalled Echo (FSPGR) T1-weighted (T1w) sequence as 50 frames over 8 min with 0.2 ml/kg contrast agent (Dotarem, 279.3 mg/ml, Guerbet, Villepinte, France) injected intravenously.

Collection of PSMA-PET data was initiated 60 min after intravenous injection of 2.0 MBq/kg [^68^Ga]PSMA-11 (median injected activity: 163 MBq; range: 121–201 MBq), lasted for 40 min, and was finalized during the MRI acquisition. The acquisition was performed from one bed position capturing the pelvic region. PSMA-PET images were reconstructed using 3D ordered subset expectation maximization with resolution recovery (SharpIR; GE Healthcare, Waukesha, WI, USA).

Patients received intravenous injection of 5 MBq/kg [^11^C]Acetate (median injected activity: 426 MBq; range: 286–544 MBq) 10 min before Acetate-PET/CT image acquisition. Data were acquired on a PET/CT system (Discovery 690; GE Healthcare, Waukesha, WI, USA) with a low-dose CT for attenuation correction followed by a diagnostic quality CT. PET-data were collected in a region spanning from the proximal femur to the head using time-of flight, 11 slices overlap, 2 min/bed position. Attenuation-corrected images were reconstructed using the SharpIR reconstruction algorithm into a 70 cm field-of-view (24 subsets, 3.0 mm post filter) [29, 30].

### Ex-vivo imaging and histopathological evaluation

Resected prostates were placed in 3D printed molds, tailored to each patient based on delineations of prostate on T2w MRI [31]. Molds containing prostates were imaged to yield ex-vivo T2w images and subsequently prepared for histopathological evaluation. Prostate specimens were fixed in formalin, sectioned to 5 mm blocks, then dehydrated and paraffin-embedded. From each block, 5 µm thick microtome sections were taken, matching the ex-vivo slices. The microtome sections were initially evaluated by a board-certified pathologist (A.B.) and subsequently digitized (NanoZoomer-XR C12000, Hamamatsu Photonics, Hamamatsu, Shizuoka, Japan). A.K.L (PhD) provided detailed digital annotations of regions with Gleason scores and internal Gleason patterns (GPs), under A.B’s supervision and final approval.

### Co-registration

T2w images were used as a common frame of reference when co-registering histopathology, PSMA‑PET/mpMRI, and Acetate‑PET/CT. Histopathological information was registered by first aligning histology slices with ex-vivo T2w MRI with the use of a 2D-affine registration method. This was followed by rigid and deformable 3D-registrations of the ex-vivo image stack to the in-vivo image. The transforms associated with the registrations were then applied to the histopathological information. Acetate-PET/CT data were rigidly registered to the T2w in-vivo image, whereas deformable registration procedures were applied for the DCE and DWI images [29, 31]. Image processing was performed in Hero v.2024.1.0 (Hero Imaging AB, Umeå, Sweden) unless otherwise stated.

### Delineation procedures

Prostate gland, genitals, and urethra (6 mm in diameter [32]) were manually delineated on T2w images. Seminal vesicles were generated from the registered CT images using the SeminalVes deep learning segmentation model of RayStation [33]. The MVision AI Contour+ (v1.2.7, Helsinki, Finland) auto-segmentation toolbox was used to segment lymph nodes, bladder, rectum, femoral heads, penile bulb, and bowel bag on CT images. In slices where the prostate or seminal vesicles appeared, we manually contoured the rectum and bladder on T2w images, overriding the CT-based delineations. We then removed the auto-segmented slices within 5 mm above and below the manually contoured region and interpolated between the two sets of slices to create the final volume.

Four radiation oncologists (observers) independently delineated gross tumor volume (GTV) on T2w, DWI, DCE, and PSMA-PET separately [19]. In line with the results of Grefve et al., GTVs for each patient and observer were formed by taking the union of the modality-specific delineations and cropping the result to the prostate gland expanded by 1 mm. The prostate clinical target volume (CTV) was defined as the whole prostate gland, including the GTV. We simulated the use of rectal spacers (Barrigel, Palette Life Sciences, Santa Barbara, CA) by overriding part of the rectal volume to ensure at least 10 mm separation from the prostate CTV. We refer to this adjusted rectal volume, excluding the volume occupied by the spacer, as rectum. The margin from the CTV to the prostate planning target volume (PTV) was 5 mm. A second CTV was constructed for pelvic lymph nodes, and 7 mm was added to form the corresponding PTV. Similarly, a third CTV was produced for the seminal vesicles (2.0 cm proximal) and an 8 mm margin was added to form the PTV.

### Protocol design and optimization strategy

**Figure 1:**
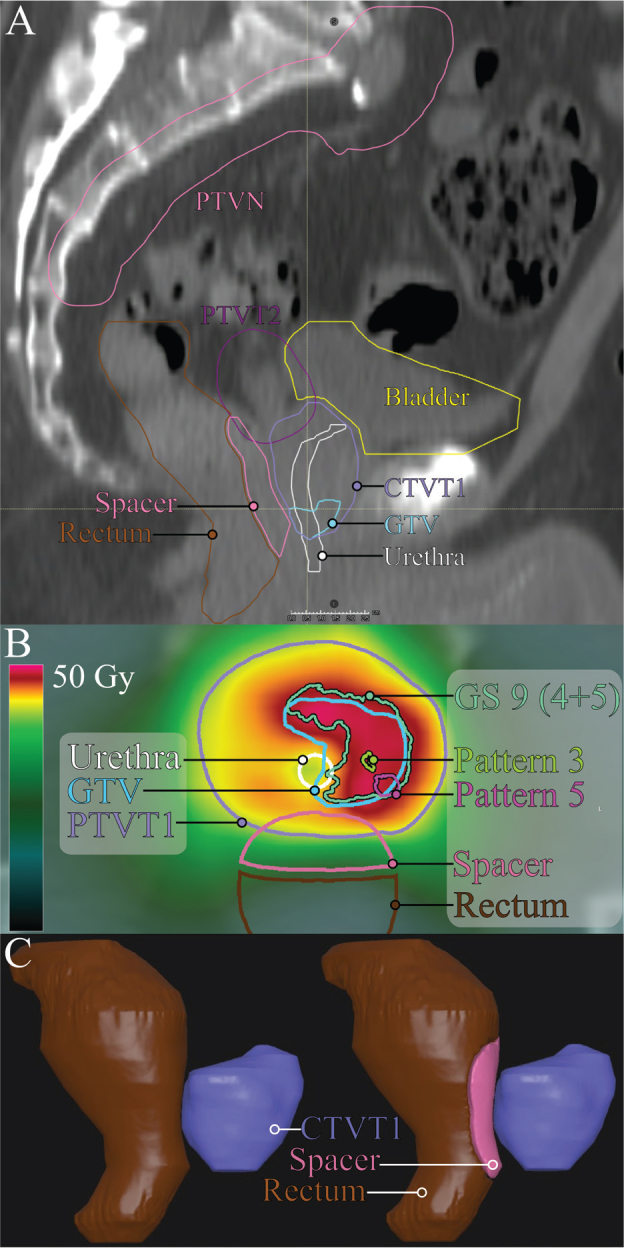
Subset of the structures used for optimization and evaluation. (A) Sagittal view of the rectal spacer, organs at risk (urethra, rectum and bladder), gross tumor volume (GTV), prostate clinical target volume (CTVT1), and planning target volumes for regional lymph nodes (PTVN) and seminal vesicles (PTVT2). (B) Axial representation of the dose distribution with contours for the urethra, GTV, prostate planning target volume (PTVT1), rectal spacer, rectum, and registered histopathology, indicating the Gleason score (GS) along with secondary and minor Gleason Patterns. (C) The rectal spacer was simulated by modifying the original rectum to create a 10 m separation from CTVT1. The horizontal dashed line in (A) corresponds with the slice shown in (B).

The protocol implemented in this study was based on the HYPO-RT-PC-boost phase II trial (NCT06220435). In brief, triple-arc 10 MV VMAT plans were generated in RayStation v.2023B (RaySearch Laboratories AB, Stockholm Sweden), with a prescription dose to the prostate PTV of 42.7 Gy in 7 fractions. The GTV was prescribed up to 49.0 Gy, prioritizing constraints for OARs. Lymph node PTV was planned to receive 29.4 Gy and seminal vesicles PTV 31.2 Gy. Additional details on planning objectives are provided in Supplementary Table 1, which also outlines how optimization volumes were generated to resolve potential conflicts between objectives. Figure 1 exemplifies an optimized treatment plan, together with targets, OARs, and registered WMHP.

### Statistical analysis

DVH analysis was performed to evaluate the planned treatments. Fleiss’ kappa [34] was used to quantify interobserver agreement in GTV delineations, and correlation analysis was performed using the Spearman correlation coefficient. All p-values less than 0.05 were deemed statistically significant.

## Results

The optimization resulted in 60 treatment plans, one for each patient and observer. The median fraction of histologically confirmed tumor also defined as GTV was estimated to be 0.61 (0.48–0.77) [19].

**Figure 2:**
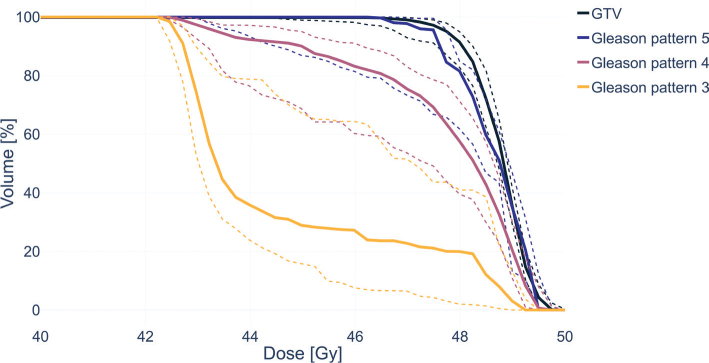
Dose-volume histograms for gross-tumor volume (GTV) and regions of Gleason patterns 3–5. The percentages of volume receiving dose (y-values) were determined per patient and observer. Solid lines represent median values over patients for all observers, and dashed lines the inter-quartile ranges.

The GTV coverage was consistent for all 60 treatment plans, with a median dose to GTV in the range 48.3–49.1 Gy. For voxels within regions of GP 5, present in three patients (12 plans), the median dose varied between 42.9 and 49.2 Gy and was at least 47.2 Gy in all except one plan. Conversely, the median dose for voxels within regions of GP 3 was below 43.4 Gy in more than half of the plans and ranged between 42.5 and 49.2 Gy. DVHs for GTVs and all GPs are shown in Figure 2. Complementary dose parameters are summarized in Table 2.

**Table 2:** Dose to 2, 50 and 98% of volume and mean dose for gross-tumor volume (GTV) and regions of Gleason patterns 3–5. Values are medians (IQR) across patients and observers.

Region	D_2_ (Gy)	D_50_ (Gy)	D_mean_ (Gy)	D_98_ (Gy)
GTV	49.6 (49.4–49.8)	48.8 (48.7–48.9)	48.8 (48.6–48.8)	47.3 (46.1–47.7)
Pattern 5	49.4 (49.2–49.6)	48.8 (48.3–48.9)	48.6 (47.9–48.7)	46.9 (43.2–47.5)
Pattern 4	49.5 (49.2–49.7)	48.3 (47.4–48.6)	47.7 (46.5–48.1)	42.8 (42.6–43.3)
Pattern 3	49.0 (48.2–49.3)	43.4 (43.0–47.1)	44.6 (43.6–46.8)	42.5 (42.3–42.6)

**Figure 3:**
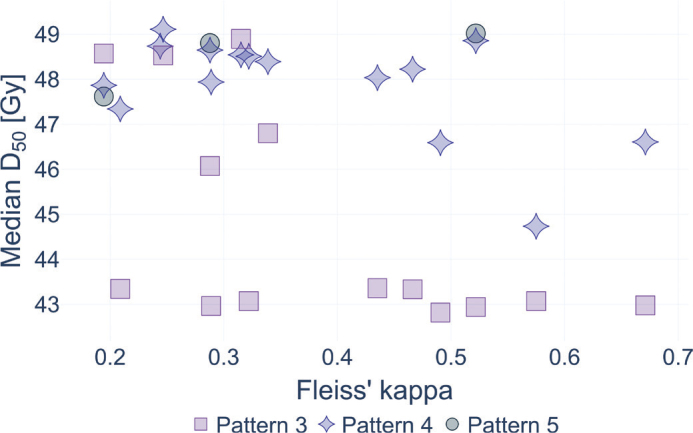
Median (across observers) D50 (median dose) per patient, for voxels within regions of Gleason pattern 3, 4, and 5, over interobserver agreement, quantified using Fleiss’ kappa.

No significant positive correlations were found between the DVH parameters presented in Table 2 (D_2_, D_50_, D_mean,_ D_98_) to regions of GP 3 and 4 and Fleiss’ kappa between observer delineations for each patient (Supplementary Figure 1 and Supplementary Tables 2–3). For regions of GP 5, the median (across observers) D_mean_ and D_50_ showed significant positive associations with Fleiss’ kappa (Figure 3 and Supplementary Table 2). However, none of the DVH parameters showed a significant positive correlation with Fleiss’ kappa across all individual observers (Supplementary Table 3–4).

Table 3 provides a comparison of dose parameters with the hypo-FLAME study [15, 17]. The narrower range of GTV doses in the present study is a consequence of optimizing for uniform dose, whereas the hypo-FLAME study applied no explicit uniformity constraint, aside from limiting hotspots. Another notable difference is the approximately 30% lower rectal doses (EQD2), resulting from our use of rectal spacers.

**Table 3:** Median (IQR) across all patients and observers. The equivalent dose in 2 Gy fractions (EQD2) is provided for each median dose level.

Parameter	Present study		hypo-FLAME[15, 17]	
	Median. [IQR]	EQD2^†^	Median. [IQR]	EQD2^†^
Urethra D_0.04 cc_ (Gy)	44.4 [43.8–44.6]	83.1	39.3 [38.6–39.9]	85.4
Bladder D_1 cc_ (Gy)	43.4 [43.2–43.5]	79.7	36.1 [35.6–37.1]	73.8
Rectum D_1 cc_ (Gy)	32.0 [30.3–32.5]	48.4	35.0 [34.8–35.4]	70.0
GTV D_mean_ (Gy)	48.8 [48.6–48.8]	118.0	44.7 [43.0–46.9]	133.3
GTV D_99_ (Gy)	47.0 [44.9–47.5]	110.3	40.3 [39.3–42.9]	110.1
GTV Volume (cc)	2.5 [1.8–3.6]	–	2.3 [0.7–4.2]	–

† Assuming α/β = 1.5 for prostate and α/β = 3.0 for risk organs. [15]

## Discussion and conclusion

This study demonstrates that hypofractionated RT for PCa with focal boost can deliver higher dose levels to sites containing more aggressive disease, while respecting OAR constraints. However, the ability to provide focal dose escalation to all malignant tissue is limited by several factors: the inherent limitations of medical imaging in depicting malignant regions, an incomplete understanding of the correlations between imaging characteristics and histological features, and the need to spare OARs. Importantly, our results indicate that observer agreement on tumor localization did not correlate well with dose coverage. These findings suggest that the role of observer consensus is unclear in settings where GTV coverage is compromised in favor of OAR constraints. Although significant positive correlations were found between dose coverage and interobserver agreement for regions of IGG 5, there were only three such datapoints, and the resulting Spearman correlations should therefore be interpreted with caution.

Several planning studies have been carried out to dosimetrically evaluate target coverage and sparing of OARs, including histologically defined boost volumes [35] and biologically driven treatment planning [36, 37]. However, there are few comparable simulation studies, tracking the dose distribution with a gold standard histopathological reference. Zamboglou et al. [23, 24] explored the feasibility of dose escalation with models for tumor control probability (TCP) and normal tissue complication probabilities (NTCP) using WMHP. Initially, GTVs were defined by PSMA-PET/CT data [23], and later they compared GTVs from PSMA-PET/CT with mpMRI-derived GTVs and combined information from the two modalities (*n* = 10) [24]. The dose escalation strategies yielded higher TCP with minimal to no increase of NTCP. However, it was beyond their scope to present how the planned dose varied over histological grades, and the TCP models were based on a constant cellular density parameter.

One principal difference between the optimization protocol in current work and that of the hypo-FLAME study is our use of rectum spacers. This approach reduced dose to rectum, making urethra dose levels the primary limitation to GTV coverage. The spacer thickness of 10 mm is in accordance with the Barrigel user guidelines (Barrigel, Palette Life Sciences, Santa Barbara, CA). Given the reported small change in spacer structure and low resorption over the timeframe of treatment [38], we re-optimized treatment plans at 8 mm spacer thickness and ensured that the evaluation criteria for rectum were met (Supplementary Tables 5 and 6).

The implemented study protocol differs from the HYPO-RT-PC-boost protocol in a few aspects. In the present study, the boost volume was formed by taking the union of modality-specific delineations instead of a single GTV based on PSMA-PET/mpMRI. This decision was in part based on data availability, where modality-specific delineations were obtained from the work of Grefve et al. [19]. The definition of GTVs will inevitably affect the coverage of histologically confirmed disease. A union-based approach was adopted to be inclusive of histologically confirmed disease, while maintaining methodological transparency and avoiding bias toward any single observer or modality. As there is a lack of established guidelines, we find support for the adopted approach by the similarity in GTV size between the current study and the hypo-FLAME trial (Table 3). Furthermore, there were no PTVs generated for the GTVs. By adopting a union-based approach, we are likely to achieve a larger boost volume compared to what would have been generated by joint PET/mpMRI [20, 24], thereby mitigating the discrepancy.

The data collection was initiated before the introduction of PI-RADS v2.1 [39]. Consequently, the image quality is likely lower than current standards, which may have affected the accuracy of delineations. However, the size of GTVs and the proportion of histologically confirmed tumor also defined as GTV are consistent with findings reported in other studies [17, 19, 20]. The small size of the cohort limits the ability to draw definitive conclusions about the dose distribution across GPs in a general population. However, by incorporating multiple observers, the study provides valuable insight into how variability in delineations impacts dose distribution across grades of PCa. Moreover, the dose analysis across GPs becomes more challenging when histological components are scattered throughout the lesion, rather than confined to distinct regions. In our approach, the majority pattern was assigned to the portion of the lesion not designated as a secondary or minority pattern. Consequently, in one patient with a post-RP IGG 5/Gleason score 9 (4+5) lesion, the entire lesion was considered as GP 4. Excluding this patient form the analysis had only minor effects on the results (Supplementary Figure 3).

This study can aid in the interpretation of results from previous studies. Our data suggest that focal dose escalation can achieve better coverage in more aggressive sites, despite differences in GTV delineations. However, true tumor was only partially boosted, and coverage of histologically confirmed tumor correlated poorly with GTV coverage (exemplified in Supplementary Figure 2). This indicates that the relationship between treatment outcomes and measures of target coverage may be less direct than is typically presented. A potential interpretation of the observed benefits from focal boosts to MR-visible tumors [40] is therefore that the dose escalation is less dependent on the exact location, implying that OARs could be given higher priority in optimization protocols.

In conclusion, in the present study the histologically confirmed tumor was only partially boosted, and observer agreement on tumor localization did not correlate well with dose coverage in regions of histologically confirmed disease. This observation likely reflects limitations of imaging methods in tumor representation, and the inability to fully exploit available image information, rather than inadequacies in dose planning.

Despite differences in GTV delineations, more aggressive sites obtained better coverage. A potential interpretation of the encouraging results of clinical trials exploring focal boost for localized PCa is that the exact location of the dose escalation may not be critical to the treatment outcome. This implies that additional concessions in favour of OARs could be considered during treatment planning, and that attempts to explain clinical outcomes using dose coverage of visible tumor alone may neglect important factors.

## Supplementary Material



## Data Availability

The data that support the findings of this work are stored in an institutional repository and can be made available from the corresponding author upon reasonable request.
