# Supplementary material for: Ultra-hypofractionated radiotherapy with focal boost for high-risk localized prostate cancer (HYPO-RT-PC-boost): in silico evaluation with histological reference
Source: Acta Oncol. 2025 Oct 27;64:44211. doi: 10.2340/1651-226X.2025.44211 (PMC12576695; doi:10.2340/1651-226X.2025.44211)
Supplement: Supplementary file 1 [file AO-64-44211-s1.pdf]

Supplementary material has been published as submitted. It has not been copyedited, or typeset by Acta Oncologica

Supplementary Information

Image processing was performed in Hero v.2024.1.0 (Hero Imaging AB, Umeå, Sweden). Statistical analysis was performed using the SciPy (v.1.15.2)[1] and Statsmodels (0.14.4)[2] packages of Python (v.3.12.0). Treatment planning was performed in RayStation v.2023B (RaySearch Laboratories AB, Stockholm Sweden).

Supplementary Table 1 – Planning objectives. External = any point within the patient; PTVT1_42.7 = prostate planning target volume (PTV), planned to receive 42.7 Gy; Y_Rectum[-1mm] = part of rectum more than 1 mm from any PTV. PTVN_29.4 = PTV for regional lymph nodes (planned to receive 29.4 Gy); PTVT2_31.2 = PTV for seminal vesicles (planned to receive 31.2 Gy); Z_GTVT3_49.0 = the gross tumor volume (GTV) minus the [urethra + 2 mm]; Y_BowelBag[-5mm] = bowelbag minus [the joint volume of all PTVs expanded by 5 mm]; Y_Bladder[-5mm] = bladder minus [the joint volume of all PTVs expanded by 5 mm]; Y_Rectum[-1mm] = Rectum minus [the volume of all PTVs expanded by 1 mm], where rectum itself excludes the volume occupied by the spacer that ensures 10 mm distance to the prostate clinical target volume; Z_PTVN_29.4[-4mm] = PTVN_29.4 minus the [the joint volume of all other PTVs expanded by 4 mm. Urethra = prostatic urethra.

Region of interest	Description	Weight (range)
External	Dose fall-off [H]43.70 Gy [L]21.35 Gy. Low dose distance 1.20 cm	800–900
External	Dose fall-off [H]43.70 Gy [L]9.00 Gy. Low dose distance 4.00 cm	800–900
PTVT1_42.7	Max DVH 42.70 Gy to 10.00 cm³ volume	100–100
PTVT1_42.7	Min dose 42.70 Gy	1000–1750
Y_Rectum[-1mm]	Dose fall-off [H]43.70 Gy [L]21.35 Gy. Low dose distance 0.70 cm	50–50
Y_Rectum[-1mm]	Dose fall-off [H]43.7 Gy [L]16.00 Gy. Low dose distance 1.00 cm	50–50
PTVN_29.4	Min dose 29.4 Gy	1000–1900
PTVN_29.4	Max DVH 30.00 Gy to 20.00 cm³ volume	80–80
PTVT2_31.2	Min dose 31.20 Gy	800–800
Z_GTVT3_49.0	Uniform dose 49.00 Gy	400–600
PTVT2_31.2	Max DVH 32.00 Gy to 15.00 cm³ volume	80–80
Y_BowelBag[-5mm]	Max EUD 9.50 Gy, Parameter A 1	30–50
Y_Bladder[-5mm]	Max EUD 22.00 Gy, Parameter A 1	30–50
Y_Rectum[-1mm]	Max EUD 13.00 Gy, Parameter A 1	30–50
Z_PTVN_29.4[-4mm]	Max dose 31.25 Gy	30–70
Urethra	Max dose 44.90 Gy	1750–2000

Supplementary Table 2 – Significant correlations ($p<0.05$) between dose statistics (median over observers for each patient) to groups of voxels of varying Gleason patterns and Fleiss' kappa between observer delineations. Data is plotted in Supplementary Figure 1.

Dose parameter	Gleason pattern	Spearman rho (p-value)
D_2	4	-0.57 (p=2.72e-02)
D_2	3	-0.69 (p=6.54e-03)
D_{50}	5	1.00 (p=0.00e+00)
D_{50}	3	-0.67 (p=8.12e-03)
D_{mean}	5	1.00 (p=0.00e+00)
D_{mean}	3	-0.67 (p=8.70e-03)
D_{98}	3	-0.54 (p=4.70e-02)

Supplementary Table 3 – Significant correlations ($p<0.05$) between dose statistics to groups of voxels of varying Gleason patterns and Fleiss' kappa between observer delineations for the four observers.

Observer	Dose parameter	Pattern	Spearman rho (p-value)
A	D_2	5	-1.00 (p=0.00e+00)
B	D_2	5	1.00 (p=0.00e+00)
B	D_2	4	-0.69 (p=4.19e-03)
A	D_2	3	-0.78 (p=8.90e-04)
B	D_2	3	-0.63 (p=1.65e-02)
C	D_2	3	-0.70 (p=5.63e-03)
D	D_2	3	-0.53 (p=4.92e-02)
B	D_{50}	5	1.00 (p=0.00e+00)
D	D_{50}	5	1.00 (p=0.00e+00)
A	D_{50}	4	-0.61 (p=1.64e-02)
A	D_{50}	3	-0.64 (p=1.38e-02)
B	D_{50}	3	-0.63 (p=1.65e-02)
C	D_{50}	3	-0.74 (p=2.45e-03)
C	D_{mean}	5	1.00 (p=0.00e+00)
D	D_{mean}	5	1.00 (p=0.00e+00)
A	D_{mean}	3	-0.74 (p=2.45e-03)
B	D_{mean}	3	-0.70 (p=5.63e-03)
C	D_{mean}	3	-0.67 (p=8.70e-03)
C	D_{98}	3	-0.73 (p=2.92e-03)

Supplementary Table 4 – Significant correlations ($p<0.05$) between dose statistics to regions of any Gleason pattern and Fleiss' kappa between observer delineations for the four observers.

Observer	Dose parameter	Spearman rho (p-value)
A	D_{50}	-0.61 (p=1.64e-02)
B	D_2	-0.67 (p=6.13e-03)

Supplementary Table 5 – Clinical goals of treatment plans using a 10 mm spacer. Results per observer (A, B, C, D). Percentage of fulfilled clinical goals over all observers and patients.

Prio	ROI	Clinical goal	A	B	C	D	Fullfilled
1	CTVT1_42.7	At least 41.80 Gy dose at 99.00 % volume	41.95–42.45 Gy	41.90–42.38 Gy	41.97–42.42 Gy	41.82–42.38 Gy	100%
2	PTVT1_42.7	At least 40.60 Gy dose at 98.00 % volume	40.92–41.84 Gy	41.18–41.88 Gy	41.10–41.89 Gy	41.15–42.07 Gy	100%
3	PTVT1_42.7	At least 39.70 Gy dose at 99.50 % volume	39.67–41.21 Gy	40.09–41.43 Gy	40.03–41.34 Gy	40.13–41.55 Gy	100%
4	Z_PTVT1_42.7[-4mm]	At most 44.80 Gy dose at 2.00 % volume	44.53–45.70 Gy	44.48–45.55 Gy	44.57–45.55 Gy	44.47–45.85 Gy	88%
5	Urethra	At most 44.80 Gy dose at 0.05 cm³ volume	43.40–45.17 Gy	43.26–44.78 Gy	43.40–44.75 Gy	43.30–45.29 Gy	98%
6	Rectum(10mm)	At most 44.80 Gy dose at 0.50 cm³ volume	24.82–34.23 Gy	25.76–34.35 Gy	25.89–34.59 Gy	25.26–34.42 Gy	100%
7	Bladder	At most 44.80 Gy dose at 0.50 cm³ volume	43.26–44.03 Gy	43.18–44.12 Gy	43.25–44.11 Gy	43.24–44.01 Gy	100%
8	GTVT3_49.0	At least 48.50 Gy dose at 99.00 % volume	42.21–48.19 Gy	43.04–48.17 Gy	42.09–48.06 Gy	42.46–48.26 Gy	15%
9	GTVT3_49.0	At least 45.60 Gy dose at 99.50 % volume	41.97–48.16 Gy	42.83–48.09 Gy	41.78–48.01 Gy	42.33–48.25 Gy	70%
10	GTVT3_49.0	At least 46.60 Gy dose at 98.00 % volume	42.45–48.28 Gy	43.33–48.23 Gy	42.49–48.18 Gy	42.69–48.31 Gy	75%
11	GTVT3_49.0	At most 51.50 Gy dose at 2.00 % volume	49.26–50.54 Gy	49.33–50.36 Gy	49.29–51.39 Gy	49.24–50.32 Gy	100%
12	Rectum(10mm)	At most 15.00 % volume at 25.00 Gy dose volume	0.62–13.77 %	0.97–13.84 %	1.06–13.87 %	0.75–13.93 %	100%
13	CTVT2	At least 30.60 Gy dose at 98.00 % volume	30.86–31.19 Gy	30.79–31.09 Gy	30.69–31.06 Gy	30.76–31.11 Gy	100%
14	CTVN	At least 28.80 Gy dose at 98.00 % volume	29.15–29.42 Gy	29.16–29.47 Gy	29.15–29.40 Gy	29.16–29.39 Gy	100%
15	Rectum(10mm)	At most 10.00 % volume at 30.00 Gy dose volume	0.02–8.76 %	0.02–8.80 %	0.11–8.92 %	0.02–8.79 %	100%
16	PTVT2	At least 29.60 Gy dose at 98.00 % volume	30.50–30.98 Gy	30.44–30.96 Gy	30.41–30.98 Gy	30.41–30.97 Gy	100%
17	PTVT2	At least 29.00 Gy dose at 99.50 % volume	29.26–30.73 Gy	29.39–30.72 Gy	29.25–30.66 Gy	29.34–30.75 Gy	100%
18	PTVN	At least 27.90 Gy dose at 98.00 % volume	28.10–28.32 Gy	28.12–28.35 Gy	28.09–28.32 Gy	28.09–28.31 Gy	100%
19	PTVN	At least 27.30 Gy dose at 99.50 % volume	27.48–27.71 Gy	27.45–27.77 Gy	27.41–27.72 Gy	27.40–27.74 Gy	100%
20	Bladder	At most 34.00 Gy average dose	16.32–32.35 Gy	16.39–31.85 Gy	16.38–32.16 Gy	16.32–32.30 Gy	100%
21	Bladder	At most 50.00 % volume at 36.00 Gy dose volume	1.63–27.29 %	1.61–25.62 %	1.61–26.07 %	1.55–26.42 %	100%
22	Rectum(10mm)	At most 28.00 % volume at 20.00 Gy dose volume	6.47–24.95 %	7.78–25.12 %	7.36–24.93 %	6.53–25.38 %	100%
23	FemoralHead	At most 29.00 Gy dose at 5.00 cm³ volume	13.62–18.67 Gy	13.23–18.03 Gy	13.54–17.98 Gy	14.11–18.30 Gy	100%
24	FemoralHead	At most 29.00 Gy dose at 5.00 cm³ volume	13.69–18.53 Gy	13.70–19.03 Gy	13.32–19.18 Gy	13.88–19.27 Gy	100%
25	PenileBulb	At most 18.00 Gy average dose	1.44–11.88 Gy	1.44–11.77 Gy	1.45–11.57 Gy	1.45–11.36 Gy	100%
26	PenileBulb	At most 32.00 Gy dose at 2.00 % volume	1.80–24.83 Gy	1.80–24.12 Gy	1.81–26.16 Gy	1.81–23.70 Gy	100%
27	PelvicBone	At most 70.00 % volume at 15.00 Gy dose volume	35.48–47.52 %	36.16–47.44 %	36.55–47.96 %	36.15–47.80 %	100%
28	PelvicBone	At most 22.00 Gy average dose	12.07–15.72 Gy	12.05–15.67 Gy	12.00–15.80 Gy	12.02–15.76 Gy	100%

29	BowelBag	At most 450.00 cm³ volume at 16.00 Gy dose	220.29–868.40 cm³	221.31–871.93 cm³	219.26–852.81 cm³	218.10–877.98 cm³	47%
30	BowelBag	At most 195.00 cm³ volume at 25.00 Gy dose	136.22–435.44 cm³	137.39–437.83 cm³	136.99–439.37 cm³	135.00–437.30 cm³	13%
31	Y_BowelBag[-5mm]	At most 25.00 cm³ volume at 30.00 Gy dose	0.00–8.42 cm³	0.00–8.14 cm³	0.00–8.41 cm³	0.00–8.45 cm³	100%
32	Y_BowelBag[-5mm]	At most 100.00 cm³ volume at 27.00 Gy dose	0.06–16.12 cm³	0.08–16.22 cm³	0.06–18.27 cm³	0.05–15.98 cm³	100%
33	Y_BowelBag[-5mm]	At most 180.00 cm³ volume at 24.00 Gy dose	5.78–44.94 cm³	5.10–46.66 cm³	5.37–47.62 cm³	5.06–44.57 cm³	100%
34	Y_BowelBag[-5mm]	At most 300.00 cm³ volume at 21.00 Gy dose	27.28–131.54 cm³	27.12–131.34 cm³	26.04–127.63 cm³	25.10–132.24 cm³	100%
35	Y_Bladder[-5mm]	At most 15.00 % volume at 32.00 Gy dose volume	0.51–12.71 %	0.52–11.42 %	0.48–13.63 %	0.52–14.69 %	100%
36	Y_Bladder[-5mm]	At most 40.00 % volume at 28.00 Gy dose volume	2.41–49.91 %	2.37–39.59 %	2.14–47.19 %	2.51–50.46 %	95%
37	Y_Genital[-7mm]	At most 5.00 % volume at 29.00 Gy dose volume	0.00–0.00 %	0.00–0.00 %	0.00–0.00 %	0.00–0.00 %	100%
38	Y_Genital[-7mm]	At most 35.00 % volume at 22.00 Gy dose volume	0.00–0.21 %	0.00–0.28 %	0.00–0.21 %	0.00–0.21 %	100%
39	Y_Genital[-7mm]	At most 50.00 % volume at 14.00 Gy dose volume	0.00–1.10 %	0.00–1.03 %	0.00–0.84 %	0.00–0.80 %	100%
40	Z_PTVT2_31.2[-4mm]	At least 32.80 Gy dose at 2.00 % volume	33.40–36.29 Gy	33.40–35.94 Gy	33.26–36.00 Gy	33.13–36.31 Gy	100%
41	Z_PTVN_29.4[-4mm]	At least 30.90 Gy dose at 2.00 % volume	31.49–32.50 Gy	31.52–32.32 Gy	31.51–32.14 Gy	31.38–32.27 Gy	100%
42	External[-PTVs]	At most 44.80 Gy dose at 0.50 cm³ volume	42.19–43.07 Gy	42.05–43.06 Gy	42.14–43.19 Gy	42.22–42.95 Gy	100%
43	z_GTVT3_49.0	At least 48.50 Gy dose at 99.00 % volume	45.68–48.19 Gy	45.52–48.17 Gy	45.56–48.06 Gy	46.11–48.26 Gy	15%
44	z_GTVT3_49.0	At least 45.60 Gy dose at 99.50 % volume	45.32–48.16 Gy	45.39–48.09 Gy	45.28–48.01 Gy	45.72–48.25 Gy	100%
45	z_GTVT3_49.0	At least 46.60 Gy dose at 98.00 % volume	46.01–48.28 Gy	45.79–48.23 Gy	45.80–48.18 Gy	46.49–48.31 Gy	93%

CTVT1_42.7 = Prostate clinical target volume; PTVT1_42.7 = Prostate target volume; Z_PTVT1_42.7[-4mm] = Optimization volume for PTVT1, removing [GTVT3_49.0 + 4 mm] ; Urethra = Prostatic urethra with diameter 6 mm; Rectum(10mm) = The anatomical rectum minus the spacer material that ensures 10 mm separation to CTVT1; GTVT3_49.0 = Visible tumor on PSMA-PET/mpMRI; CTVT2 = Seminal vesicles clinical target volume; PTVT2 = Seminal vesicles planning target volume; PTVN = Lymph node planning target volume; Y_BowelBag[-5mm] = Optimization volume for bowel bag, determined by subtracting from Bowelbag the combined volume occupied by all other planning target volumes expanded by 5 mm; Y_Genital[-7mm] = Optimization volume for genitals, generated analogously to Y_BowelBag; Y_Bladder[-5mm] = Optimization volume for bladder, analogous to Y_BowelBag; Z_PTVT2_31.2[-4mm] = Optimization volume for PTVT2, subtracting from PTVT2 [PTVT1 + 4 mm]; External[-PTVs] = Volume not occupied by any planning target volume; z_GTVT3_49.0 = Optimization volume for GTVT3, generated by subtracting from GTVT3 the urethra expanded by 2 mm.

Supplementary Table 6 – Clinical goals of treatment plans using an 8 mm spacer. Results per observer (A, B, C, D). Percentage of fulfilled clinical goals over all observers and patients.

Prio	Region of interest	Clinical goal	A	B	C	D	Fullfilled
1	CTVT1_42.7	At least 41.80 Gy dose at 99.00 % volume	42.03–42.40 Gy	41.92–42.34 Gy	41.85–42.49 Gy	42.02–42.40 Gy	100%
2	PTVT1_42.7	At least 40.60 Gy dose at 98.00 % volume	41.13–41.85 Gy	41.18–41.86 Gy	41.16–41.98 Gy	41.11–41.97 Gy	100%
3	PTVT1_42.7	At least 39.70 Gy dose at 99.50 % volume	39.92–41.18 Gy	40.15–41.21 Gy	40.06–41.57 Gy	40.12–41.50 Gy	100%
4	Z_PTVT1_42.7[-4mm]	At most 44.80 Gy dose at 2.00 % volume	44.47–45.73 Gy	44.52–45.90 Gy	44.61–45.48 Gy	44.63–46.04 Gy	85%
5	Urethra	At most 44.80 Gy dose at 0.05 cm³ volume	43.35–45.01 Gy	43.37–44.73 Gy	43.40–44.74 Gy	43.21–45.05 Gy	100%
6	Rectum(10mm)	At most 44.80 Gy dose at 0.50 cm³ volume	24.91–35.05 Gy	24.62–34.82 Gy	25.60–34.87 Gy	24.69–34.80 Gy	100%
7	Bladder	At most 44.80 Gy dose at 0.50 cm³ volume	43.30–44.08 Gy	43.30–44.28 Gy	43.22–44.23 Gy	43.24–44.03 Gy	100%
8	GTVT3_49.0	At least 48.50 Gy dose at 99.00 % volume	42.06–48.04 Gy	42.98–48.15 Gy	41.78–48.12 Gy	42.28–48.35 Gy	10%
9	GTVT3_49.0	At least 45.60 Gy dose at 99.50 % volume	41.66–48.03 Gy	42.79–48.04 Gy	41.52–48.11 Gy	42.12–48.33 Gy	67%
10	GTVT3_49.0	At least 46.60 Gy dose at 98.00 % volume	42.24–48.10 Gy	43.24–48.25 Gy	42.17–48.18 Gy	42.52–48.44 Gy	75%
11	GTVT3_49.0	At most 51.50 Gy dose at 2.00 % volume	49.35–51.21 Gy	49.22–50.87 Gy	49.11–50.59 Gy	49.27–50.77 Gy	100%
12	Rectum(10mm)	At most 15.00 % volume at 25.00 Gy dose volume	0.65–13.19 %	0.61–13.13 %	0.91–13.14 %	0.56–13.18 %	100%
13	CTVT2	At least 30.60 Gy dose at 98.00 % volume	30.85–31.07 Gy	30.82–31.25 Gy	30.81–31.06 Gy	30.81–31.17 Gy	100%
14	CTVN	At least 28.80 Gy dose at 98.00 % volume	29.15–29.39 Gy	29.15–29.34 Gy	29.10–29.37 Gy	29.16–29.41 Gy	100%
15	Rectum(10mm)	At most 10.00 % volume at 30.00 Gy dose volume	0.02–8.75 %	0.02–8.82 %	0.07–8.74 %	0.02–8.81 %	100%
16	PTVT2	At least 29.60 Gy dose at 98.00 % volume	30.55–30.97 Gy	30.53–30.98 Gy	30.53–30.97 Gy	30.51–30.99 Gy	100%
17	PTVT2	At least 29.00 Gy dose at 99.50 % volume	29.28–30.70 Gy	29.22–30.74 Gy	29.38–30.75 Gy	29.21–30.74 Gy	100%
18	PTVN	At least 27.90 Gy dose at 98.00 % volume	28.07–28.32 Gy	28.13–28.31 Gy	28.11–28.29 Gy	28.07–28.32 Gy	100%
19	PTVN	At least 27.30 Gy dose at 99.50 % volume	27.44–27.67 Gy	27.41–27.73 Gy	27.47–27.69 Gy	27.35–27.69 Gy	100%
20	Bladder	At most 34.00 Gy average dose	17.04–31.60 Gy	16.42–31.67 Gy	16.73–31.65 Gy	16.58–31.68 Gy	100%
21	Bladder	At most 50.00 % volume at 36.00 Gy dose volume	1.60–25.67 %	1.67–25.79 %	1.55–26.39 %	1.67–26.54 %	100%
22	Rectum(10mm)	At most 28.00 % volume at 20.00 Gy dose volume	6.30–23.64 %	5.78–24.53 %	6.49–23.59 %	5.53–24.44 %	100%
23	FemoralHead	At most 29.00 Gy dose at 5.00 cm³ volume	13.88–18.61 Gy	14.15–18.35 Gy	13.19–18.78 Gy	13.99–18.19 Gy	100%
24	FemoralHead	At most 29.00 Gy dose at 5.00 cm³ volume	14.13–18.85 Gy	14.02–19.35 Gy	13.60–18.94 Gy	13.97–18.80 Gy	100%
25	PenileBulb	At most 18.00 Gy average dose	1.44–11.15 Gy	1.45–11.90 Gy	1.37–11.69 Gy	1.44–11.63 Gy	100%
26	PenileBulb	At most 32.00 Gy dose at 2.00 % volume	1.79–25.69 Gy	1.80–23.58 Gy	1.67–26.17 Gy	1.79–25.04 Gy	100%
27	PelvicBone	At most 70.00 % volume at 15.00 Gy dose volume	37.43–47.89 %	36.65–47.13 %	37.32–45.80 %	37.29–46.06 %	100%
28	PelvicBone	At most 22.00 Gy average dose	12.19–15.75 Gy	12.12–15.65 Gy	12.08–15.69 Gy	12.13–15.66 Gy	100%

29	BowelBag	At most 450.00 cm³ volume at 16.00 Gy dose	229.50–887.38 cm³	227.19–874.57 cm³	231.03–880.21 cm³	229.14–878.55 cm³	47%
30	BowelBag	At most 195.00 cm³ volume at 25.00 Gy dose	137.73–443.12 cm³	137.59–437.89 cm³	137.02–438.63 cm³	135.58–438.10 cm³	13%
31	Y_BowelBag[-5mm]	At most 25.00 cm³ volume at 30.00 Gy dose	0.00–8.54 cm³	0.00–8.21 cm³	0.00–8.43 cm³	0.00–8.56 cm³	100%
32	Y_BowelBag[-5mm]	At most 100.00 cm³ volume at 27.00 Gy dose	0.04–17.41 cm³	0.09–15.47 cm³	0.13–15.62 cm³	0.01–15.61 cm³	100%
33	Y_BowelBag[-5mm]	At most 180.00 cm³ volume at 24.00 Gy dose	5.18–48.48 cm³	6.00–43.86 cm³	4.90–44.81 cm³	5.96–44.20 cm³	100%
34	Y_BowelBag[-5mm]	At most 300.00 cm³ volume at 21.00 Gy dose	28.59–139.04 cm³	28.14–129.65 cm³	27.78–128.60 cm³	27.82–131.10 cm³	100%
35	Y_Bladder[-5mm]	At most 15.00 % volume at 32.00 Gy dose volume	0.45–12.16 %	0.63–12.19 %	0.47–13.49 %	0.60–13.11 %	100%
36	Y_Bladder[-5mm]	At most 40.00 % volume at 28.00 Gy dose volume	2.20–36.46 %	2.47–39.32 %	2.88–34.35 %	2.53–39.00 %	100%
37	Y_Genital[-7mm]	At most 5.00 % volume at 29.00 Gy dose volume	0.00–0.00 %	0.00–0.00 %	0.00–0.00 %	0.00–0.00 %	100%
38	Y_Genital[-7mm]	At most 35.00 % volume at 22.00 Gy dose volume	0.00–0.14 %	0.00–0.19 %	0.00–0.17 %	0.00–0.21 %	100%
39	Y_Genital[-7mm]	At most 50.00 % volume at 14.00 Gy dose volume	0.00–0.88 %	0.00–1.38 %	0.00–0.82 %	0.00–1.08 %	100%
40	Z_PTVT2_31.2[-4mm]	At least 32.80 Gy dose at 2.00 % volume	33.34–36.07 Gy	33.43–36.24 Gy	33.44–36.27 Gy	33.68–36.24 Gy	100%
41	Z_PTVN_29.4[-4mm]	At least 30.90 Gy dose at 2.00 % volume	31.47–32.15 Gy	31.39–32.23 Gy	31.42–32.24 Gy	31.45–32.15 Gy	100%
42	External[-PTVs]	At most 44.80 Gy dose at 0.50 cm³ volume	42.31–43.24 Gy	42.20–42.91 Gy	42.30–43.17 Gy	42.27–43.20 Gy	100%
43	z_GTVT3_49.0	At least 48.50 Gy dose at 99.00 % volume	44.64–48.04 Gy	45.31–48.15 Gy	45.15–48.12 Gy	45.53–48.35 Gy	10%
44	z_GTVT3_49.0	At least 45.60 Gy dose at 99.50 % volume	44.44–48.03 Gy	45.05–48.04 Gy	44.70–48.11 Gy	45.30–48.33 Gy	95%
45	z_GTVT3_49.0	At least 46.60 Gy dose at 98.00 % volume	45.27–48.10 Gy	45.76–48.25 Gy	45.36–48.18 Gy	46.14–48.44 Gy	95%

Rectum(8mm) = The anatomical rectum minus the spacer material that ensures 8 mm separation to CTVT1. See Table 5 for descriptions of the remaining regions of interest.

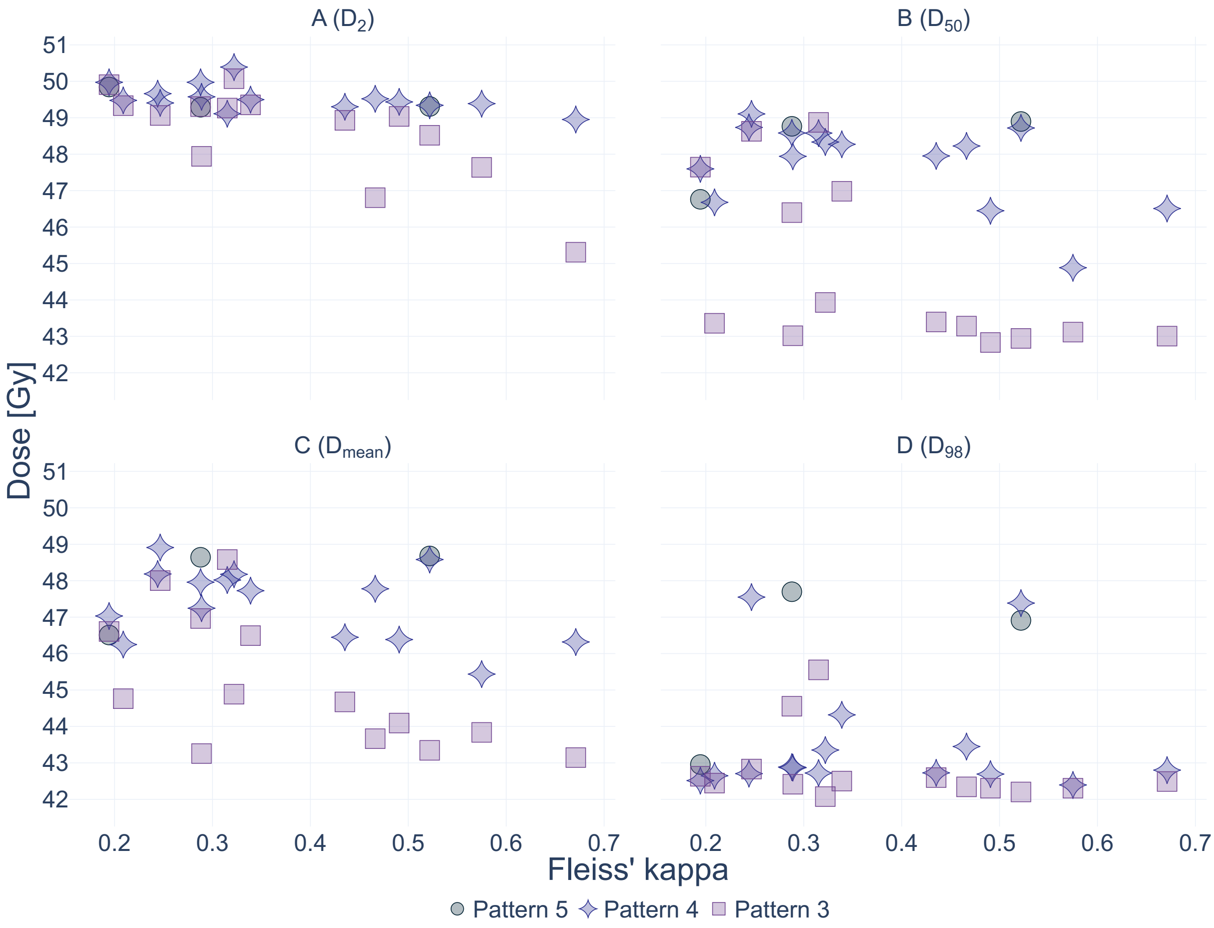

Supplementary Figure 1 – DVH measures per patient (median over observers) for groups of voxels of varying Gleason pattern, over interobserver agreement, quantified using Fleiss' kappa between observer delineations. (A) Near-minimum dose (D_2), (B) median dose, (C) mean dose and (D) Near-minimum dose (D_{98}).

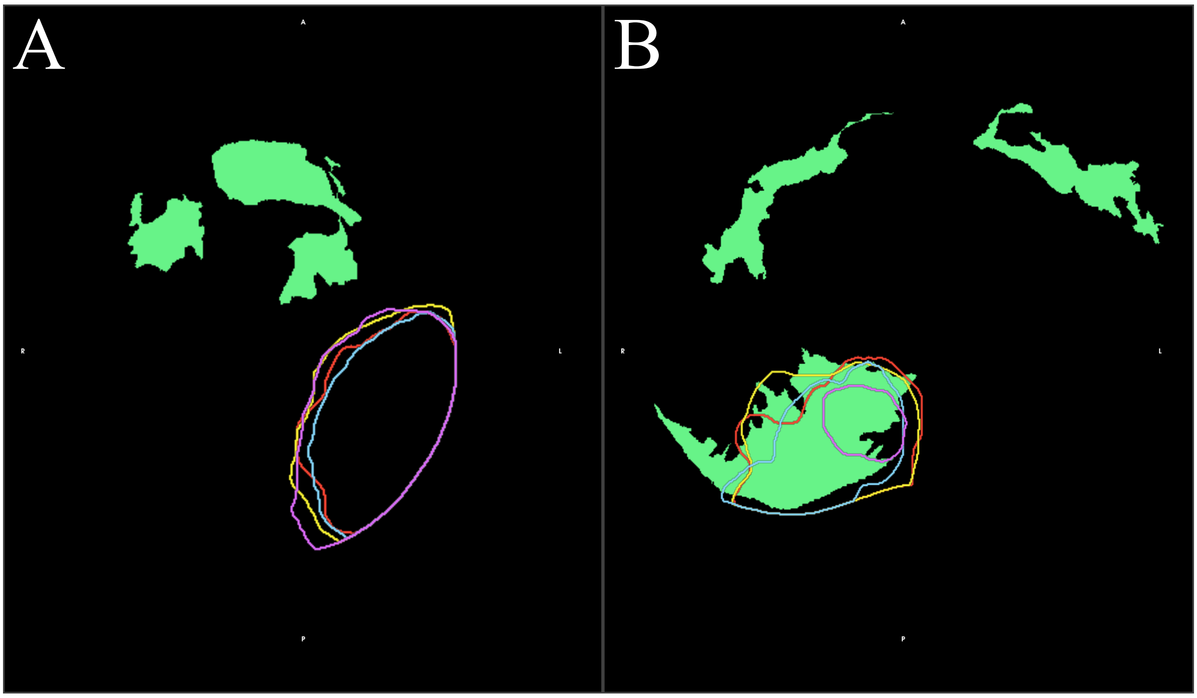

Supplementary Figure 2 – Exemplifying the observation that observer agreement correlates poorly with target coverage. Histologically confirmed lesions regions are shown as filled areas in green, and the four observer delineations are shown as colored outlines.

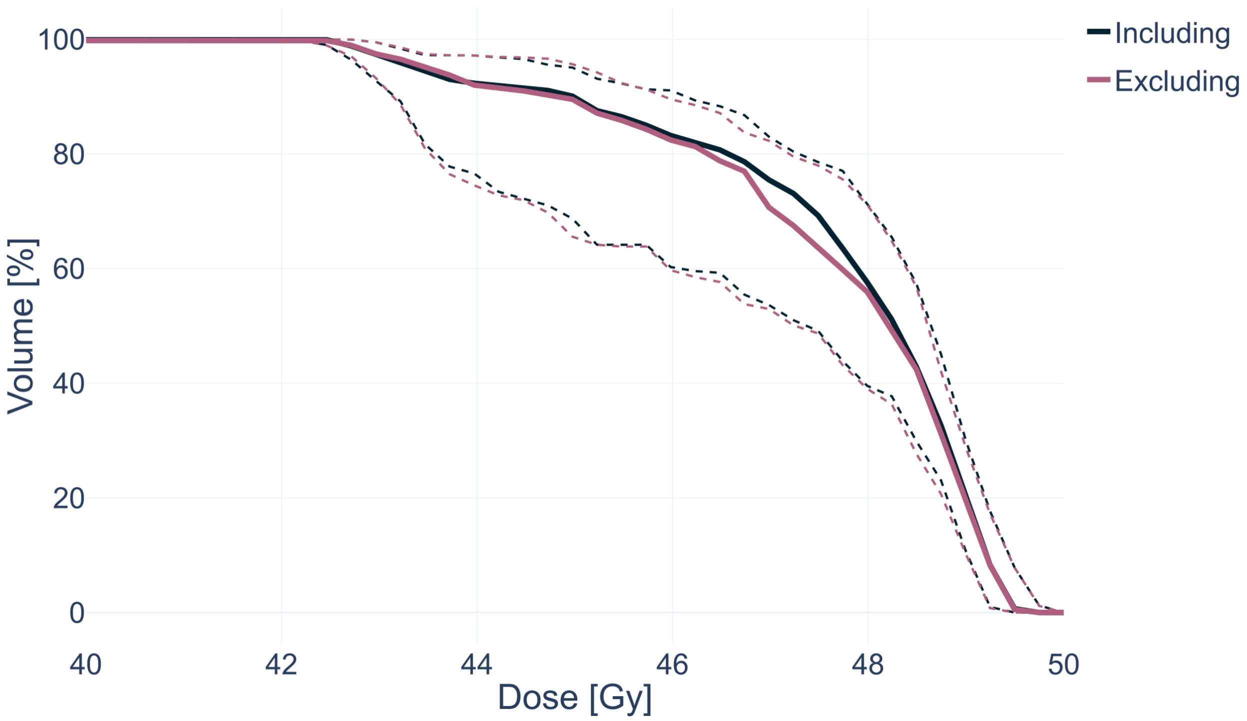

Supplementary Figure 3 – Dose-volume histograms for regions of Gleason pattern 4 when including (black) or excluding (red) a Gleason score 9 (4+5) lesion, where the secondary Gleason pattern 5 could not be demarcated and the whole lesion was considered Gleason pattern 4. The percentages of volume receiving dose (y-values) were determined per patient and observer. Solid lines represent median values over patients for all observers, and dashed lines the inter-quartile ranges.

1. Gommers R, Virtanen P, Haberland M, Burovski E, Reddy T, Weckesser W, et al., *SciPy 1.15. 0.* Zenodo, 2024.
2. Seabold S and Perktold J, *Statsmodels: econometric and statistical modeling with python.* SciPy, 2010. 7(1): p. 92-96.
